# A systematic review on shared biological mechanisms of depression and anxiety in comorbidity with psoriasis, atopic dermatitis, and hidradenitis suppurativa

**DOI:** 10.1192/j.eurpsy.2021.2249

**Published:** 2021-11-25

**Authors:** Michele Fabrazzo, Salvatore Cipolla, Simona Signoriello, Alessio Camerlengo, Giulia Calabrese, Giulia Maria Giordano, Giuseppe Argenziano, Silvana Galderisi

**Affiliations:** 1 Department of Psychiatry, University of Campania Luigi Vanvitelli, 80138 Naples, Italy; 2 Medical Statistics Unit, Department of Mental Health and Public Medicine, University of Campania Luigi Vanvitelli, 80138 Naples, Italy; 3 Dermatology Unit, University of Campania Luigi Vanvitelli, 80131 Naples, Italy

**Keywords:** Anxiety, chronic inflammatory skin diseases, depression, shared biologic mechanisms

## Abstract

**Background:**

Mental disorders in comorbidity with chronic skin diseases may worsen disease outcome and patients’ quality of life. We hypothesized the comorbidity of depression, anxiety syndromes, or symptoms as attributable to biological mechanisms that the combined diseases share.

**Methods:**

We conducted a systematic review based on the Preferred Reporting Items for Systematic Review and Meta-Analysis statement searching into PubMed, PsycInfo, and Scopus databases. We examined the literature regarding the comorbidity of psoriasis (Ps), atopic dermatitis (AD), or hidradenitis suppurativa with depression and/or anxiety in adults ≥18 years and the hypothetical shared underlying biological mechanisms.

**Results:**

Sixteen studies were analyzed, mostly regarding Ps and AD. Brain-derived neurotrophic factor/tropomyosin receptor kinase B signaling and nuclear factor kappa-light-chain-enhancer of activated B cells/p38 mitogen-activated protein kinase pathways arose as shared mechanisms in Ps animal models with depression- and/or anxiety-like behaviors. Activated microglia and neuroinflammatory responses emerged in AD depressive models. As to genetic studies, atopic-dermatitis patients with comorbid anxiety traits carried the short variant of serotonin transporter and a polymorphism of the human translocator protein gene. A GA genotype of catechol-O-methyltransferase gene was instead associated with Ps. Reduced natural killer cell activity, IL-4, serotonin serum levels, and increased plasma cortisol and IgE levels were hypothesized in comorbid depressive AD patients. In Ps patients with comorbid depression, high serum concentrations of IL-6 and IL-18, as well as IL-17A, were presumed to act as shared inflammatory mechanisms.

**Conclusions:**

Further studies should investigate mental disorders and chronic skin diseases concurrently across patients’ life course and identify their temporal relation and biological correlates. Future research should also identify biological characteristics of individuals at high risk of the comorbid disorders and associated complications.

## Introduction

Common mental disorders, such as depression and anxiety, show a high prevalence in patients with chronic inflammatory processes underlying physical diseases. Indeed, the incidence of psychiatric disorders is higher in patients with chronic immune-mediated inflammatory diseases when compared with the general population. For example, depression is present in up to 50% of patients with chronic systemic conditions (e.g., pain, stroke, cardiovascular disease, obesity, diabetes, and cancer), compared with 5–8% in the general population [1]. Depression and anxiety are common in patients affected by rheumatoid arthritis, the prototype of inflammatory arthritis, with 13.4% diagnosed with anxiety and 41.5% with depression [2]. In patients with chronic skin disorders, a prevalence rate of 10.1% for depression and 17.2% for anxiety was reported compared with healthy subjects [3].

Furthermore, comorbid mental symptoms or syndromes may negatively affect physical diseases and exacerbate a patient’s psychopathology, worsening disease outcome and quality of life (QoL), besides life expectancy [3–6].

Several studies over the last 20 years showed that the hyperactivity of the hypothalamic–pituitary–adrenal (HPA) axis and the sympathetic nervous system (SNS) is the most reported mechanism that might underlie the association with chronic inflammatory diseases and mood disorders [7].

Loftis et al. [8] and Myint et al. [9] suggested a causal association between chronic treatments with specific cytokines and severe side effects involving the immune and brain system in patients with malignancies or chronic immune-mediated diseases. In particular, a greater risk of developing a major depressive episode within 3 months after the initial treatment with interferon-alpha (IFN-α) was reported in about 39% of the initially euthymic patients with no history of psychiatric disorders within the past 6 months. Chronic inflammation and depression are also associated with increased corticotropin-releasing hormone and related glucocorticoid receptor resistance, which, in turn, contribute to the increase in inflammatory cytokines and persistence of inflammation [10–14]. The excessive, enduring cytokine production may also activate brain microglia, a mechanism supposedly underlying depression, anxiety, and cognitive impairment [15].

A hypothesis recently formulated suggests that the inflammasome protein complex and related inflammatory reactions would act as the triggering mechanism of the reciprocal/bidirectional relationship between the stress-related psychiatric illness and comorbid systemic disease [16]. According to this hypothesis, the inflammasome complex is a central mediator by which psychological and physical stressors might contribute to developing depression and, as well, act as a bridge to systemic diseases. Scientific research has mainly concentrated on the impact of stress on the relationship between brain and peripheral systems and on which type of biological factors might be involved as potential mediators.

Growing evidence supports the modulatory role of immune responses leading to cytokine-mediated inflammatory responses, the activation of the predominant stress pathways as the HPA axis, resulting in the cortisol release, and the activation of the SNS, which leads to the epinephrine and norepinephrine release [17–19].

Investigating the time sequence of biological mechanisms that produce the comorbidity of depression and/or anxiety with inflammatory skin diseases might contribute to determining their causal relation. In addition, identifying shared mechanisms of mental disorders and comorbid chronic inflammatory disorders might improve their treatment and outcome.

Skin diseases entailing chronic inflammatory processes, such as psoriasis (Ps), atopic dermatitis (AD), and hidradenitis suppurativa (HS), are notably debilitating disorders associated with a heavy psychological burden [6,20–22]. Indeed, psychiatric comorbidity is estimated to affect over 30% of patients with dermatologic disorders [23].

The recent literature suggested that several mechanisms activated by chronic inflammation may also act as common mediators in the comorbid process [24]. The long-established explanatory model of causation under which disability and related physical symptoms cause mental health problems is debated. Scientific evidence showed complex interrelations based on which mental disorders and physical diseases can reciprocally originate and lead to increased severity of both. Such a bidirectional relationship may precipitate or exacerbate both mental and physical symptoms in vulnerable individuals. Consequently, shared inflammatory mechanisms may function as the biological link between physical diseases and mental disorders.

A few papers reported the activation of proinflammatory cytokines in the pathogenesis of chronic inflammatory skin diseases, such as Ps, AD, and HS [25,26]. In Ps, an upregulation of the IL-17 axis emerged as well as an excess of IL-12, IL-23, and TNF-α [26]. Skin biopsies from AD patients show a predominance of T-helper-2-derived cytokines, IL-4, and IL-13 [27]. Recent effective targeted therapy directed at the IL-4 and IL-13 axis demonstrated the importance of this pathway in AD [28]. Instead, the cytokine signature in HS may include dysregulation of TNF, IL-1, IL-12/23, IL-17, and IL-6 [29].

Furthermore, patients with comorbid chronic skin inflammatory diseases and mental symptoms exhibited drastic behavioral changes, long recognized as distinguishing features of the “sickness behavior” [30,31]. Such features encompass fatigue, insomnia, anorexia, and loss of interest in the social and physical environment, representing common features of depressive and anxiety disorders. It has been suggested that the behavioral changes are mediated by proinflammatory cytokines released by the activated immune cells [15,32]. Therefore, such symptoms may display as a maladaptive cytokine-induced “sickness behavior” due to the persistent or excessive activation of the immune response or stem from the propensity to depression or anxiety in the presence of an inadequate immune response [15].

Several clinical studies reported comorbid depression in up to 42% of patients with HS [33] and 60% in patients with Ps [21]. Mental symptoms are described as related to chronic inflammatory skin diseases due to psychosocial factors and impaired QoL. Among such factors, the most typical are anxiety and depression associated with feelings of stigmatization and consequent social withdrawal, insomnia due to impelling itch, and body image disorders associated with the severity of skin lesions [23]. In particular, epidemiological studies evidenced that patients with chronic inflammatory skin disorders manifest a significant prevalence of anxiety and depression, and conversely, anxiety and depression increase the prevalence of chronic inflammatory skin disorders [34]. In addition, patients with AD and HS may experience severe symptoms of a depressive condition, such as elevated suicidal ideation [35,36]. Currently, it proves difficult to determine whether mental symptoms in patients with chronic diseases, such Ps, AD, and HS, result from the underlying inflammatory processes or the psychosocial burden of a chronic illness in subjects vulnerable to common mental disorders [26,36]. In addition, psychological factors may exacerbate inflammatory cutaneous diseases or even trigger adverse reactions to conventional therapies [37].

In conclusion, further extensive studies are decisive to uncover the pathogenesis of chronic skin diseases comorbidity with psychiatric disorders, especially cellular and biochemical pathways. In addition, such comorbidity throughout a patient’s life might enable clinicians to identify the biological correlates and their time relation to achieve novel treatment targets.

Researchers should detect the mechanisms under which most patients with depression or anxiety disorders do not develop a chronic skin inflammatory disease, and vice versa, and identify the biological characteristics of individuals at high risk and complications.

The present review article provides an overview of the evidence based on the hypothesis that the frequent comorbidity of depression, anxiety symptoms or syndromes, and chronic inflammatory skin diseases is attributed to biological mechanisms that such diseases share.

We conducted a scoping review of the clinical- and population-based literature relevant to the comorbidity of Ps, AD, or HS and depression and/or anxiety in adults ≥18 years and the hypothetical shared underlying biological mechanisms.

We hypothesize that the comorbidity pathogenesis includes several elements that might activate or suppress biological pathways, that is, the release of neurotransmitters, specific proinflammatory cytokines and trophic factors, immune system responses through activated immune cells, and release of antibodies, which may produce the dysfunction of the nervous system and peripheral organ systems.

## Methods

This systematic review will be reported according to the Preferred Reporting Items for Systematic Review and Meta-Analysis (PRISMA) statement, as applicable [38].

The items of the review protocol will be the following:

1. Stage 1: Identify the research questions.
2. Stage 2: Identify the relevant studies.
3. Stage 3: Study selection process.
4. Stage 4: Chart the data.
5. Stage 5: Collate, summarize, and report the results.

### Stage 1: Identify the research questions

Our research questions were developed on the model of the existing reviews that described the prevalence of mental disorders or symptoms, the influence of psychosocial factors on the disease’s outcome, and the related impairment of patients’ QoL [3,6,25]. Furthermore, the past reviews also investigated the effects of biologic drugs in dermatologic patients with a skin disease entailing chronic inflammatory processes, such as Ps, AD, and HS, concurrent with psychiatric disorders [39–41]. The existing literature primarily focuses on Ps patients [42], thus, only limited data are available for AD and HS. Furthermore, some authors identified inflammatory mechanisms as a possible link between depression and/or anxiety and chronic skin diseases. In particular, the hypothesized bidirectional relationship and the cytokine involvement underlying the comorbid conditions, reported in the literature, need to be elucidated to explain the connection between the immune and brain system, besides the neuroendocrine system and the behavioral alterations in dermatologic patients with comorbid affective disorders. In addition, several studies produced evidence of a significant improvement of psychopathological symptoms due to treatment with biological agents [43], indirectly suggesting that such drugs might act on shared biological mechanisms of the comorbid conditions.

In the light of the above considerations, we examined studies including patients ≥18 years diagnosed with one of the three chronic skin disorders (Ps, AD, and HS) appearing in comorbidity with mental syndromes or symptoms.

We aimed to answer the following research questions:

1. Which biological mechanisms may explain the comorbidity between chronic skin disorders (Ps, AD, and HS) and mood disorders/symptoms in patients whose primary diagnosis was a dermatologic disease?
2. What are the gaps in the literature regarding this topic?
3. Which suggestions can we give for future research?

### Stage 2: Identification of relevant studies

#### Eligibility criteria

We identified depression and anxiety as the mental disorders of interest for this scoping review and also included depressive/anxiety symptoms assessed through standardized psychopathological rating scales, independent of the presence of a clear-cut anxiety or depressive disorder based on DSM-5 criteria.

We included all randomized controlled trials (RCTs), cross-sectional, case-control, and cohort studies on patients ≥18 years with a clinical or histological diagnosis of chronic skin disorders, namely Ps, AD, and HS, in concurrence with depression and/or anxiety. We included only studies that adopted a standardized measure for depression and/or anxiety (such as a psychopathological rating scale). Furthermore, we included as well studies on patients treated with biologic drugs currently approved to treat Ps, AD, and HS. In particular, we examined studies in which TNF-α inhibitors, IL-17 inhibitors, IL-12/23 inhibitors, anti-IgE antibody, and anti-CD20 were used to treat the skin disorders we selected for our analysis. In addition, we included only studies that reported a 12-week follow-up, the lapse of time often needed to resolve long-lasting depressive symptoms.

We also included basic research studies reporting on biological mechanisms analyzed in animals in which the corresponding human skin disorders were artificially induced, and the resulting depression- and anxiety-like behaviors were analyzed by appropriate tests.

Finally, we excluded studies that presented confounding effects of major concomitant physical illnesses (e.g., recent myocardial infarction or malignancy).

#### Outcomes

We selected all the studies reporting on comorbidity between chronic skin disorders (namely Ps, AD, and HS) and depression and/or anxiety based on the evaluation severity of both conditions through reported outcome measures. Thus, our primary outcome will be evaluating the shared mechanisms reported as biological markers of the two conditions.

#### Search strategy

We searched on PubMed, PsycInfo, and Scopus databases for relevant reviews, case reports, case series, and pharmacologic trials performed on animals and humans, with English as a language filter. Date limits were from inception to May 2021. Search terms (MeSH headings) included (“Biological Factors”[MeSH Terms] OR “Inflammation”[MeSH Terms] OR “Immune System Phenomena”[MeSH Terms] OR “Amino Acids, Peptides, and Proteins”[MeSH Terms]) AND (“Depression”[MeSH Terms] OR “Depressive Disorder”[MeSH Terms] OR “Affective Symptoms” [MeSH Terms] OR “Anxiety Disorders”[MeSH Terms] OR “Anxiety”[MeSH Terms]) AND (“Hidradenitis”[MeSH Terms] OR “Psoriasis”[MeSH Terms] OR “dermatitis, atopic”[MeSH Terms]) AND (“loattrfull text”[Filter] AND “English”[Language]).

In addition, we hand-searched the reference lists of included articles for any additional relevant studies.

### Stage 3: Selection of studies

The titles and abstracts of articles were initially screened for inclusion by M.F. and G.C. using our predetermined criteria. Selected articles were later assessed independently and unblinded by G.M.G., S.C., S.S., and G.C. Any disagreement between the reviewers was resolved by consensus.

### Stage 4: Data extraction

S.C. and A.C. extracted the relevant data, which were subsequently synthesized in a tabular format; G.M.G., S.S., and G.A. triple-checked extracted data for accuracy; M.F., S.C., and A.C. extracted data on study characteristics (participant age and sex, sample size, and dermatologic diagnostic tools), outcome measures (proportion of patients with depression and/or anxiety symptoms as defined by study investigators, and psychopathological assessment tools used to evaluate the severity of depression and/or anxiety), and therapeutical intervention type when applicable.

### Stage 5: Collation, summary, and report of results

The search strategy and selection process results will be presented in figures (flowchart) and text. The description of all the studies selected will be presented as both text and tables.

In addition, the main results will be summarized for each research question, and study limitations, literature gaps, and areas needing further investigation will be highlighted.

## Risk of Bias Assessment of the Studies Examined in the Review

Two review authors (S.C. and A.C.) assessed the risk of bias of the nonrandomized studies of interventions (NRSIs) independently through the risk of bias tool (ROBINS-I) [44], based on the following seven domains:

1. bias due to confounding;
2. bias in selecting the study participants;
3. bias in classifying interventions;
4. bias due to deviating from the intended intervention;
5. bias due to missing data;
6. bias in measuring outcomes; and
7. bias in selecting reported results.

The studies were judged as being at high, moderate, and low risk of bias or no information for all domains. The two authors resolved disagreements through discussion or involving a third author (S.S.). In line with the ROBINS-I tool, the authors considered an NRSI as at low risk if judged at low risk of bias for all domains; at moderate risk if judged at moderate risk for at least one domain; at high risk if judged at high risk of bias for at least one domain but not at critical risk of bias in any domain; and at critical risk if judged at critical risk in at least one domain. We indicated “no information” for an NRSI in case no clear judgement of high or critical risk of bias was possible and in case information about one or more key domains was missing.

The same two authors (S.C. and A.C.) independently evaluated each study for the risk of bias, using the criteria recommended for RCTs in the Cochrane Handbook for Systematic Reviews of Interventions [45]. The authors judged each domain as having a low, high, or unclear risk of bias, and resolved disagreements through discussion or involving a third review author (S.S.). S.C. and A.C. evaluated the following domains in a table reporting the risk of bias:

1. sequence generation for randomization (to assess possible selection bias);
2. allocation concealment (to assess possible selection bias);
3. blinding of health professionals and participants (to assess possible performance bias);
4. blinding of outcome assessors (to assess possible detection bias);
5. incomplete outcome data (to assess possible attrition bias);
6. selective outcome reporting (to assess possible reporting bias); and
7. other potential threats to validity.

According to Higgins and Green [45], we assessed the overall risk of bias by considering how the findings were impacted by the magnitude of bias with random sequence generation, allocation concealment, incomplete outcome data, and selective reporting. We measured a low overall risk of bias if the studies used a truly random process for sequence generation, concealed allocation, had <10% of missing outcome data, and did not selectively report prespecified outcomes. Differently, we used sensitivity analysis to classify the studies as being at high risk of overall bias and to assess the impact of this risk.

## Results

**Figure 1.**
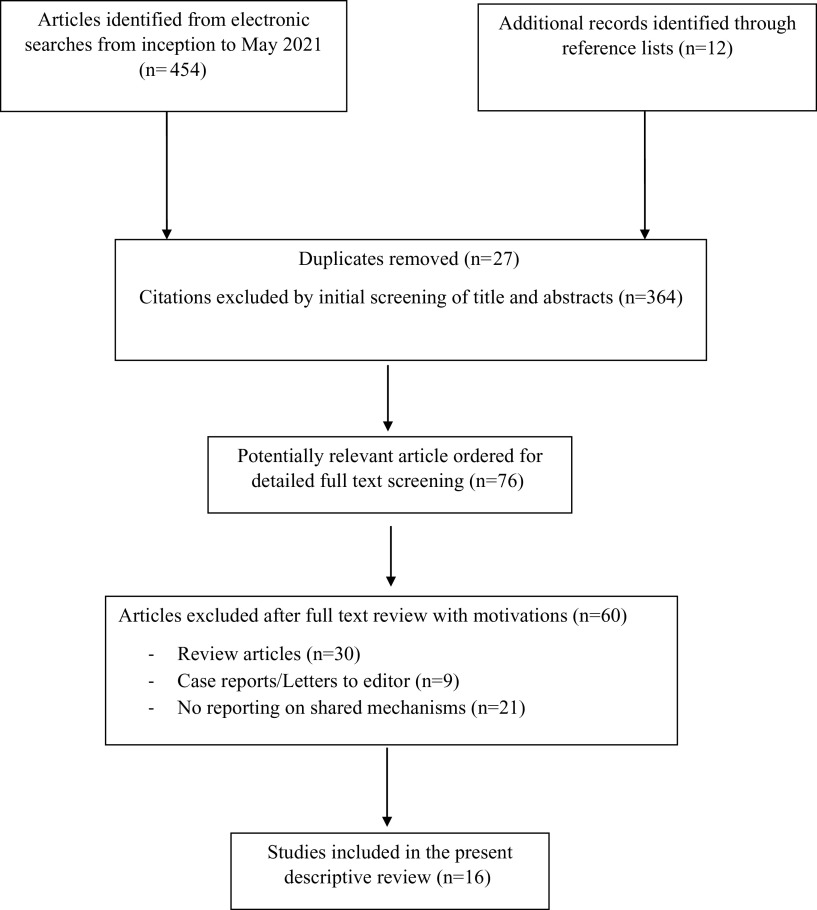
Flowchart showing study selection process of included articles.

Our literature search initially identified 466 articles (Supplementary Figures 1–3), of which only 16 were included in the final analysis. Figure 1 shows the flowchart of the included studies and illustrates the exclusion criteria we adopted. We removed 27 duplicates of studies and excluded 364 citations by initial screening of titles and abstracts. After that, we selected 76 articles potentially relevant for full-text screening, and excluded 60 of them after a careful reading: 30/60 were narrative reviews or reviews that did not analyze studies on patients with comorbid conditions or underlying potentially shared biological mechanisms, 9 were case reports or letters to editors, and 21 did not report on biological mechanisms relevant to our research questions.

Among the remaining 16 studies, 4 preclinical studies reported shared biological mechanisms in animal models of chronic skin disorders and depression, 4 genetic studies analyzed genotype distribution and allele frequencies in patients with comorbid chronic skin disorders and depression, and 8 clinical reports evaluated common biological mechanisms in patients with comorbid skin and mental disorders. The latter included 3 case-control studies and one cross-sectional report on AD patients; 3 case-control studies and one RCT on Ps patients.

### Preclinical studies

Our literature search yielded four preclinical studies: two presenting animal models for Ps and two for AD. We did not retrieve any study on HS patients (Table 1).

**Table 1.** Preclinical studies on relevant shared mechanisms of chronic skin inflammatory diseases and depression and/or anxiety in animal models.

Authors (year of publication)	Animal model	Behavioral features	Behavioral tests	Biological indices	Pharmacological probe outcomes	Mechanisms outlined	Comparison with human skin disorder
JiaWen et al. (2017)	K5.Stat3C mice, TPA-treated to induce psoriasis-like lesions	Depression/anxiety-like behaviors	FST, OFT, and EPM	BDNF and TrkB mRNA in prefrontal cortex and hippocampus.	The SSRI fluoxetine: Reduced TPA-induced skin lesions; Increased expression of BDNF and TrkB; The TrkB antagonist K252a reversed all these effects.	BDNF/TrkB signaling may participate in the mechanism of depression and anxiety behaviors in Ps.	In Ps patients, plasma BDNF concentration is decreased.
Nadeem et al. (2017)	IMQ-induced psoriasis-like skin inflammation in mice	Depression-like symptoms	TST, FST, sucrose preference test, and locomotor activity test	Phosphorylated NFκB p65 subunit and p38 MAPK in different brain regions.	IMQ treatment led to increased expression of IL-17A in innate and adaptive immune cells in different brains regions (mainly hippocampus and prefrontal cortex). Treatment with IL-17A led to depression-like behavior, reversed by anti-IL17A antibody treatment.	Systemic IL-17A has the potential to induce depression-like behavior with concurrent increase in proinflammatory pathways in the brain of mice; IL-17A-induced depression-like symptoms via the activation of NFκB and p38 MAPK pathways.	Plasma IL-17A levels proved to be elevated in Ps patients. IL-17A released from different immune cells in Ps patents may be responsible for the development of depressive symptoms.
Park et al. (2020)	DfE-induced mouse model of AD	Depressive-like behavior	Scratching of dorsal skin or ears test; OFT; Sucrose preference test	Levels of IgE, histamine, corticosterone, serotonin, MDC, TARC, and TSLP. Histopathological infiltration of inflammatory cells and mast cells into the skin and ear tissue; Elevated microglia activation and neuroinflammatory response in the brain of AD mice.	Topical application of Kudo extract: Reversed scratching behavior, ear swelling, open-field locomotion, sucrose preference, and the increased levels of IgE, histamine, cortisol, and serotonin; Reduced inflammatory cytokine responses in keratinocyte cells.	Elevated expression of TNF-α, IFN-γ, CD68, Iba-1, and NFkB in the brain; Increased serum cortisol and reduced brain 5-HT concentration.	AD is an IgE-mediated disorder where mast cells are central players in skin stress responses and are also involved in AD-related neurogenic inflammation.
Yeom et al. (2020)	MC903-induced AD-like skin lesions	Anxiety- and depressive-like behaviors	Scratching behavior, EPM, OFT, and TST	Corticosterone serum levels; Protein levels of dopamine- and plasticity-related signaling molecules measured in nucleus accumbens, dorsal striatum, and ventral tegmental area.	MC903 topical treatment: enhanced CREB, DARPP32 phosphorylation, BDNF and delta-FosB; reduced TH and D1R protein expression.	Striatal BDNF, phosphor-DARPP32, and phospho-CREB levels were associated with severity of depressive-like behavior and with activity of the reward circuitry.	In AD patients, neuroimaging studies have addressed brain system implicated in the control of reward and motivated behaviors.

*Abbreviations*: 5-HT, serotonin; AD, atopic dermatitis; BDNF, brain-derived neurotrophic factor; CD68, cluster of differentiation 68 protein; CREB, cAMP-response element binding protein; D1R, dopamine D1 receptor; DARPP-32, dopamine- and cAMP-regulated phosphoprotein, 32 kDa; Delta FosB, a truncated splice variant of the FosB gene; DfE, dermatophagoides farinae extract; EPM, elevated plus-maze test; FST, forced swimming test; Iba-1, ionized calcium binding adaptor molecule 1; IFN-γ, interferon gamma; IgE, immunoglobulin E; IL-17A, interleukin-17A; IMQ, imiquimod; MC903, calcipotriol, a synthetic VitD_3_ analogue with high affinity for vitamin D receptor; MDC, macrophage-derived chemokine; NFκB, nuclear factor kappa-light-chain-enhancer of activated B cells; OFT, open-field test; p38 MAPK, p38 mitogen-activated protein kinase; SSRI, selective serotonin reuptake inhibitor; TARC, thymus and activation-regulated chemokine; TH, tyrosine hydroxylase; TNF-α, tumor-necrosis factor-alpha; TPA, 12-O-tetradecanoylphorbol-13-acetate; TrkB, tropomyosin receptor kinase B; TSLP, thymic stromal lymphopoietin; TST, tail suspension test.

JiaWen et al. [46] reported that depression- and anxiety-like behaviors significantly increased in K5.Stat3C mice, an animal model of Ps. In addition, mRNA levels of brain-derived neurotrophic factor (BDNF) and tropomyosin receptor kinase B (TrkB) in the prefrontal cortex and the hippocampus notably decreased. The use of fluoxetine, a specific serotonin reuptake inhibitor (SSRI), not only attenuated the depression- and anxiety-like behaviors, but, in parallel, increased the expression of BDNF and TrkB. In addition, the 12-O-tetradecanoylphorbol-13-acetate-induced Ps-like pathological lesions significantly ameliorated in K5.Stat3C mice after treatment with fluoxetine. More importantly, the effects of the SSRI were reversed by K252a, an antagonist of TrkB, which suggested that BDNF/TrkB signaling was also involved in the underlying mechanism of depression- and anxiety-like behaviors in mice.

Nadeem et al. [47] hypothesized, instead, that the systemic elevated IL-17A levels reported by several studies in Ps patients may be responsible for depressive symptoms through the induced neuronal inflammatory pathways. The authors reported that in mice, the induction of psoriatic inflammation by imiquimod (an immunomodulator substance), as well as the direct administration of IL-17A, was associated with depression-like symptoms via the activation of the pathways involving both nuclear factor kappa-light-chain-enhancer of activated B cells (NFκB) and a cytokinin specific binding protein, the so-called p38 mitogen-activated protein kinase (MAPK). Moreover, monotherapy with IL-17A in naïve mice also led to depression-like symptoms. Furthermore, NFκB, as well as p38 MAPK inhibitors, attenuated IL-17A-induced depression-like symptoms in mice via the reduction in inflammatory mediators, such as the monocyte chemotactic protein-1, the inducible nitric oxide synthase, IL-6, and chemokine ligand-2. Furthermore, the anti-IL17A antibody also led to a reduction in imiquimod-induced depression-like symptoms, as well as NFκB/p38 MAPK signaling. Thus, the authors concluded that IL-17A plays an essential role in comorbid depression associated with psoriatic inflammation, where both NFκB and p38 MAPK pathways are involved via the upregulation of inflammatory mediators in the brain.

Park et al. [48] investigated the association between AD and depression in a dermatophagoides farinae extract-induced mouse model of the skin disorder.

AD mice showed more scratching behavior, increased ear swelling, and higher serum levels of IgE and histamine when compared with normal mice. AD mice also presented depressive-like behaviors in the open field, sucrose preference tests, and altered serum cortisol and brain serotonin concentrations. In addition, histopathological analyses revealed an increased infiltration of inflammatory cells and mast cells into the skin and ear tissue, as well as high activated microglia and neuroinflammatory response in the brains of AD mice. Topical application of Kudo extract improved AD-related scratching behavior, ear swelling, open-field locomotion, and sucrose preference. In addition, the Kudo extract decreased the levels of IgE, histamine, cortisol, serotonin, and inflammatory markers (as cytokine concentrations) in keratinocyte cells.

Yeom et al. [49] hypothesized that in a mouse model of AD induced by repeated intradermal application of MC903, a synthetic vitamin D_3_ analog, the anxiety- and depression-like symptoms were associated with changes in the brain reward dopaminergic circuitry, and increased plasma corticosterone levels (Table 1). Striatal BDNF, phospho-DARPP32, and phospho-CREB levels emerged as biological factors significantly associated with the severity of depressive-like behavior in mice.

### Genetic studies

Our literature search retrieved only two genetic studies on Ps patients and two on AD patients (Table 2).

**Table 2.** Studies addressing a possible genetic linkage between chronic skin inflammatory diseases and depression and/or anxiety.

Authors (year of publication)	Study population	Control population	Psychopathological evaluation	Studied genes	Conclusions
Mossner et al. (2009)	309 Ps patients (127 F and 182 M), mean age 46.0 ± 14.3 years; 140 patients with Ps positive family history.	315 healthy unrelated subjects without personal or family history of Ps (146 F and 169 M), mean age 35.6 ± 12.1 years.	HAM-D performed only in a subgroup of 137 patients (aged 18–60 years) with mild/severe depression (HAM-D > 8).	Promoter of the gene encoding SERT (5-HTTLPR)	No significant difference in genotype distribution and allele frequencies and HAM-D score in Ps patients (*n* = 137) carrying different 5-HTTLPR genotypes.
Sobolev et al. (2019)	88 Ps patients, selected according to ICD-10 criteria.	365 healthy subjects.	Clinical diagnosis.	DBH, CCKAR, CCKBR, and COMT	Polygenic analysis revealed two genotypes associated with Ps: 1. A combination of COMT heterozygote (rs4680:GA) and a deletion in DBH gene (Ins/Del 19:D); 2. COMT heterozygote (rs4680:GA).
de Mel et al. (2012)	33 patients (26 F and 7 M) with a history of AD and anxiety, mean age 39.6 ± 12.3 years; AD diagnosed according to Hanifin and Rajka criteria.	33 healthy subjects (primarily staff personnel and students), mean age 40.6 ± 12.2 years.	KSP questionnaire.	Short and long alleles of 5-HTTLPR	All AD patients with high anxiety traits carried the short variant of 5-HT transporter intron 2 polymorphism.
Kaga et al. (2014)	52 AD patients (30 M and 22 F); AD severity quantified by SCORAD Index.	163 healthy volunteers (89 M and 74 F).	STAI.	TSPO (18 kDa) gene	In AD patients, lymphocyte genomic analysis revealed an SNP of the human TSPO gene at exon 4 (485G>A); AD patients showed lower frequencies of GG and higher frequencies of GA.

*Abbreviations*: F, female; M, male; HAM-D, Hamilton Rating Scale for Depression; SERT, serotonin transporter; 5-HTTLPR, serotonin-transporter-linked promoter region; ICD-10, International Classification of Diseases, Tenth Revision; DBH, dopamine beta-hydroxylase; CCKAR, cholecystokinin A receptor; CCKBR, cholecystokinin-B receptor; COMT, catechol-O-methyltransferase; KSP, Karolinska Scales of Personality; STAI, State-Trait Anxiety Inventory; SCORAD, SCORing Atopic Dermatitis; TSPO, translocator protein.

In light of the role of T-cell-mediated inflammation and increased prevalence of depression in Ps patients, Mössner et al. [50] analyzed the polymorphism of the promoter of the gene encoding the serotonin-transporter-linked promoter region (5-HTTLPR) in patients with Ps and healthy controls. No significant difference emerged in genotype distribution and allele frequencies. In addition, no difference arose in the Hamilton Rating Scale for Depression (HAM-D) score in patients with Ps (*n* = 137) characterized by the carriage of different 5-HTTLPR genotypes. Such findings do not support a major contribution of the 5-HTTLPR polymorphism to Ps susceptibility and depressive symptoms in psoriatic patients.

Sobolev et al. [51] investigated associations of Ps with a single nucleotide polymorphism (SNP) in different genes, such as several encoding enzymes involved in the biosynthetic and catabolic pathways of some neurotransmitters such as catechol-O-methyltransferase (COMT), dopamine beta-hydroxylase (DBH), cholecystokinin-A-receptor, and CCK-B-receptor. Among the studied genes, the authors found that only a GA genotype of the COMT gene was significantly associated with Ps (*χ*^2^ = 19.163 [*p* = 1.3E−5], *F* (*p*) = 1.2E−5, OR 3.47 [CI 99% = 1.61–7.91]). The functional significance of such association remains challenging to explain. However, in line with the study conducted by Mössner et al. [50] in Ps patients, de Mel et al. [52] reported that AD patients with comorbid anxiety traits carried the short variant of 5-HT transporter intron 2 polymorphism. Kaga et al. [53], as well, reported that a lymphocyte genomic analysis in AD patients revealed an SNP of the human translocator protein (TSPO) gene at exon 4 (485G>A); AD patients showed lower frequencies of genotype GG and higher frequencies of genotype GA. The TSPO, also known as peripheral benzodiazepine receptor (PBR), is a TSPO (18 kDa), mainly found on the outer mitochondrial membrane of glial cells, extensively used as a biomarker of brain injury and inflammation [54].

### Clinical studies

The overall risk of bias was high for all the included nonrandomized clinical studies (Supplementary Table 1). Instead, the risk of bias appeared low for most items and subitems analyzed in the sole RCT included in the present review (Supplementary Table 2).

Three out of four clinical studies included case-control studies with a sample frame appropriate to address the target population—all AD patients with comorbid depression and/or anxiety versus healthy volunteers. However, the number of participants was small, and the settings were not specified. Therefore, differences between cohorts of inpatients and outpatients did not emerge. Finally, the statistical modeling of the relationships between the mental/physical symptoms and the hypothesized underlying mechanisms was inadequate to detect the complex interrelationships among the different sets of variables. Major risks of bias concerned information bias at the intervention domain (Supplementary Table 1).

Hashiro et al. [55] did not specify the dermatologic diagnostic assessment tools used to evaluate the skin disorder. Indeed, in this study, the authors did not include any information on confounding and selection bias of the pre-intervention domains, as well as on confounding bias of the post-intervention domains. In addition, the risk of bias was high as to information bias of the intervention domain (Supplementary Table 1). However, the authors reported that in AD patients with depressive symptoms and state anxiety, natural killer (NK) activity was reduced along with plasma IL-4 levels. Vinnik et al. [56], on the other hand, identified the increased plasma levels of IgE and cortisol as common pathogenetic mechanisms. The overall risk of bias for this study was high, due to a high score on confounding and information bias. These authors hypothesized that in AD male patients, especially during exacerbation of the skin disease, the increased cortisol levels may be associated with the activated HPA axis response caused by IgE-mediated degranulation of mast cells. Furthermore, Vinnik et al. revealed that dehydroepiandrosterone (DHEA), a testosterone metabolite, may be involved as a regulator of IgE synthesis and eosinophil proliferation. Finally, Jaworek et al. [57] investigated in comorbid patients solely the plasma content of 5-HT, which in depressed AD patients with a Montgomery Asberg Depression Rating Scale (MADRS) >12 was decreased as compared with healthy controls. The study of Jaworek et al. [57], though presenting a high overall risk of bias, showed a high score only on information bias of the intervention domain.

The fourth analyzed study was a cross-sectional study showing the key role of stress and anxiety in worsening AD through the serotoninergic system. Rasul et al. [58] analyzed skin biopsies in a cohort of 28 AD patients with comorbid state and somatic trait anxiety and mild depression. In lesioned skin, 5-HT immunoreactivity was high in the inflammatory infiltrate along with 5-HT1A and 5-HT2A receptors and serotonin‑selective reuptake transporter (SERT) immunoreactivity, thus confirming the involvement of serotonin in AD patients. However, this study presented an overall high risk of bias and information bias resulted at the post-intervention domains.

The three case-control studies and the RCT on Ps patients included a limited number of patients, diagnosed by dermatologic and psychopathological assessment tools. Furthermore, the abovementioned studies included an eligible cohort of patients, specifically Ps patients with comorbid depression versus controls, evaluated in an appropriate setting.

Kartha et al. [59] reported that serum melatonin levels were significantly reduced in Ps patients, compared with controls, when measured at nighttime. The study is characterized by high confounding and information bias (Supplementary Table 1). When the study population was subgrouped into Ps patients with and without depressive symptoms, no difference emerged in serum melatonin levels. Consequently, the hypothesis of reduced melatonin as a shared pathogenetic mechanism in Ps and depression was excluded. The study of Griffiths et al. [41], though presenting a high risk of bias as to “attrition bias” (incomplete outcome data addressed; Supplementary Table 2), evidenced in patients with comorbid Ps and depression the dose-dependent effects of a 12-week treatment with ixekizumab, a high-affinity monoclonal antibody selectively targeting IL-17A. Treatment with the biologic drug improved depression (33.6 and 45.2% of patients treated with ixekizumab at 80 mg/4weeks and 80 mg/2 weeks, respectively) and decreased serum levels of C-reactive protein (sCRP; 23.3 vs. 27.4% patients with sCRP levels still >5 mg/L), thus suggesting that both inflammatory markers and IL-17A may be involved in Ps comorbid depressive patients.

The study of Pietrzak et al. [60] is mainly characterized by information bias at the intervention domain, and confounding bias at the post-intervention level (Supplementary Table 1). The authors reported as common biological markers high concentrations of interleukin 18 (IL-18) and low concentrations of 25-hydroxyvitamin D_3_ (25-hydroxy-vit D_3_) associated with depression severity in men with Ps and high body mass index (BMI). Differently, Marek-Józefowicz et al. [61] identified as a shared biological mechanism the increased IL-6 plasma levels in a cohort of Ps patients with comorbid depression and specific affective temperament dimensions (Table 3).

**Table 3.** Clinical studies investigating shared biological mechanisms in patients with a chronic inflammatory skin disorder and mental symptoms/syndromes (depression and/or anxiety).

Authors (year of publication)	Study population	Dermatologic assessment	Psychiatric assessment	Biological indices	Results
Hashiro et al. (1998)	27 AD patients (mean age 24.5 ± 5.8 years) vs. 30 controls (mean age 29.5 ± 7.1 years)	Clinical diagnosis	STAI; SDS; CMI	NK cell activity; IFN-ɣ and IL-4 serum levels	Reduced NK cell activity and decreased serum IL-4 levels in moderate/severe AD patients vs. controls.
Rasul et al. (2016)	28 AD patients (18 F and 10 M; mean age 29.5 years)	SCORAD; VAS for Pruritus Intensity	SSP for anxiety; MADRS-S	5-HT, 5-HT1A-R, 5-HT2A-R, and SERT levels from lesional and nonlesional skin	High epidermal 5-HT IR in inflammatory infiltrated, HT1A-R-IR in apical epidermis, 5-HT2A-R-IR in mast cell-like cells in dermis, and SERT-IR in basal epidermal layer of lesional skin of AD patients vs. controls.
Vinnik et al. (2020)	56 AD patients (27 M and 29 F; mean age 41.6 ± 2.3 years) vs. 49 healthy controls (23 M and 26 F; mean age 34.9 ± 1.3 years)	SCORAD	HAM-D	IgE; serum cortisol and testosterone levels	Increased IgE and cortisol levels in AD males during exacerbation of the skin disease. DHEA may be one of the regulators of IgE synthesis and eosinophil proliferation in AD patients.
Jaworek et al. (2020)	31 AD patients (17 F and 14 M; median age 41 years) vs. 14 healthy controls (8 M and 6 F; median age 42 years)	Hanifin and Rajka AD criteria; SCORAD	MADRS	Plasma level of 5-HT	Decreased 5-HT plasma concentration in depressed AD patients (MADRS > 12) vs. healthy controls.
Kartha et al. (2013)	36 Ps patients vs. 36 controls	PASI	BDI	Serum melatonin	Nighttime serum melatonin levels significantly reduced in Ps patients, as compared with controls. No difference emerged in serum melatonin levels between Ps patients with or without depressive symptoms.
Griffiths et al. (2017)	Ps patients treated with: Placebo (*n* = 93; mean age 46.8 ± 13.1 years); ixekizumab 80 mg/4 weeks (*n* = 120; mean age 44.2 ± 13.3 years); ixekizumab 80 mg/2 weeks (*n* = 107; mean age 45.5 ± 12.4 years)	sPGA score ≥ 3; BSA score ≥ 10 %; PASI score ≥ 12	QIDS-SR 16	sCRP (>5 mg/L, at baseline)	In patients with comorbid Ps and depression, 12-week treatment with ixekizumab (80 mg/4 weeks or 80 mg/2 weeks) improved depression in a dose-dependent manner (33.6 and 45.2% of patients, respectively) and decreased sCRP levels (23.3% vs. 27.4 patients with sCRP levels still >5 mg/L).
Pietrzak et al. (2018)	85 Ps patients (M; aged 47 ± 14 years) vs. 65 controls (M; 44 ± 13 years)	PASI; BSA	BDI	IL-6, IL-18, cortisol, and 25-OH D_3_ plasma levels	Ps-depressed patients showed higher serum concentrations of IL-18 and lower serum 25-OH D_3_ vs. controls.
Marek-Józefowicz et al. (2021)	110 Ps patients (mean age 44.1 ± 13.1 years) vs. 98 controls (mean age 36.8 ± 10.4 years)	PASI	TEMPS-A BDI	IL-6 plasma levels	Ps-depressed patients with specific affective temperament dimensions showed higher plasma concentrations of IL-6 vs. patients without depression and vs. controls.

*Abbreviations*: DHEA, dehydroepiandrosterone; F, female; M, male; AD, atopic dermatitis; STAI, State-Trait Anxiety Inventory; SDS, Self-Rating Depression Scale; CMI, Cornell Medical Index; NK, natural killer; IFN-ɣ, interferon-gamma; IL-4, interleukin 4; SCORAD, SCORing of Atopic Dermatitis; VAS, visual analogue scale; SSP, Swedish Universities Scales of Personality; MADRS-S, Montgomery-Åsberg Depression Rating Scale—Self-assessment; 5-HT, serotonin; 5-HT1A-R, serotonin 1A receptor; 5-HT2A-R, serotonin 2A receptor; SERT, serotonin transporter; IR, immunoreactivity; HAM-D, Hamilton Rating Scale for Depression; IgE, immunoglobulin E; MADRS, Montgomery-Åsberg Depression Rating Scale; sPGA, static Physician Global Assessment; BSA, body surface area; PASI, Psoriasis Area Severity Index; QIDS-SR 16, Quick Inventory of Depressive Symptomology—Self-Report; sCRP, serum C-reactive protein; BDI, Beck Depression Inventory; IL-6, interleukin 6; IL-18, interleukin 18; 25-OH D_3_, 25-hydroxyvitamin D_3_; BMI, body mass index; TEMPS-A, Temperament Evaluation of Memphis, Pisa, Paris and San Diego-Autoquestionnaire version.

In this study, the authors did not give any information on selection bias (pre-intervention domains, confounding, and information bias in the post-intervention domains; Supplementary Table 1).

## Discussion

The present review aimed to analyze the possible pathophysiological mechanisms underlying the comorbidity of a few chronic inflammatory skin diseases (Ps, AD, and HS) and mental syndromes or symptoms (depression and anxiety) to fill the existing gaps of the literature. Recent progress must be acknowledged in research on the pathogenesis and treatment of chronic dermatologic disorders and psychiatric comorbidities.

The studies we analyzed identified several biological mechanisms as a common denominator of the postulated reciprocal/bidirectional relationship between inflammatory skin disorders and depression and/or anxiety. However, it remains unclear whether such mechanisms are the sole biological expression of peripheral cell-mediated inflammatory processes with the subsequent involvement of activated microglia, decreased neurogenesis, and increased apoptosis or a primary CNS process that extends to peripheral organs.

Furthermore, most studies have not widely explored the issue of the longitudinal course and cause relationship of the comorbid processes.

Preclinical studies suggested that BDNF/TrkB signaling may be involved in the underlying mechanisms of depression- and anxiety-like behaviors in Ps mice models [46]. In line with these findings, Ps patients with comorbid depression and/or anxiety disorders reported a decreased plasma concentration of BDNF [62,63]. BDNF also proved to be a biochemical mechanism operating in AD patients, who showed significantly higher plasma levels when compared with those of Ps patients [64,65]. Thus, the increased concentration of BDNF in AD appeared to belong to a framework of an altered neuroimmune regulatory system associated with an anxiogenic-like phenotype. Yeom et al. [49] hypothesized that the increased striatal BDNF concentration, along with phospho-DARPP32 and phospho-CREB, in a mouse AD model, was associated with the depressive-like behavior and increased activity of the dopaminergic reward circuitry, which is central in mediating anxiety, depression, and stress (Table 1).

Park et al. [48], instead, highlighted the increased infiltration of inflammatory cells and mast cells into the skin and ear tissue, as well as the elevated microglia activation and neuroinflammatory response in the brains of AD mice. Thus, the authors concluded that neuroinflammation (i.e., increased macrophage and microglial activation and expression of proinflammatory cytokines) might be a shared pathogenetic mechanism of AD and depressive-like behavior in an AD mouse model.

A second shared biological mechanism outlined by the preclinical studies in a Ps animal model is the systemic elevation of IL-17A levels, possibly triggering depressive symptoms by activating biological pathways involving both NFκB and a cytokinin-specific binding protein, the so-called p38 MAPK [45]. IL-17A appears crucial in the pathogenesis of Ps [66]. The numbers of Th17 cells and the concentration of IL-17A are reported to be much higher in psoriatic skin lesions than in healthy skin. Moreover, Th17 cells and IL-17A levels appear to be elevated in the blood of Ps patients and correlated with disease severity [67]. Elevated plasma levels of IL-17A are associated with depression and are shown to promote neuronal inflammation in both humans and animals [68,69]. Thus, the Th17 axis may be essential in neuroimmune interactions, although the role and the timing of IL-17A involvement in such interactions are not yet completely understood.

The interactive communication between the neuroendocrine and immune systems may be partly mediated by serotonin, which is involved in the physiological functions of the skin through its receptors [70,71]. Furthermore, 5-HT may also act in the inflammatory processes of the skin, including Ps [72]. A functional length polymorphism in the serotonin transporter gene promoter (5-HTTLPR) appears to be implicated in the genetic background of depression. Consequently, the serotonergic system might prove to be responsible for a neuroimmunological link between skin diseases and psychopathological symptoms. The serotonin transporter is central in the 5-HT system regulation and widely expressed on cells of the immune system, thus influencing T- and B-cell function [71]. Among genetic studies we retrieved, Mössner et al. [50] did not find a significant contribution of the 5-HTTLPR polymorphism to Ps susceptibility and comorbidity of Ps with depressive symptoms. Differently, Sobolev et al. [51], who investigated several genes encoding for enzymes involved in neurotransmitter metabolic pathways, evidenced that only a GA genotype of the COMT gene was significantly associated with Ps. Therefore, the authors excluded the 5-HT involvement and suggested a role for catecholamines and norepinephrine in particular. Such neurotransmitter increased in Ps patients’ blood [73,74] and is possibly implicated in developing inflammatory cAMP-mediated processes.

Instead, de Mel et al. [52] reported that the short variant of 5-HT transporter intron 2 polymorphism was a possible linkage between AD patients and anxiety traits.

Finally, Kaga et al. [53] reported the results of genomic analysis in AD patients that revealed an SNP of the human TSPO gene at exon 4 (485G>A). The TSPO, formerly known as PBR, is a TSPO operating mainly in glial cells, extensively used as a biomarker of inflammation [54], and involved in regulating several major stress systems, that is, the HPA axis, the SNS, the renin-angiotensin-aldosterone system, and the neuroendocrine-immune axis [75,76]. TSPO may be pivotal in the comorbidity of AD and anxiety through the synthesis of neurosteroids by promoting the cholesterol transport to the inner mitochondrial membrane, which is the rate-limiting step in neurosteroidogenesis. In addition, neurosteroids are allosteric modulators of GABA-A receptor function, crucial in the pathophysiology of anxiety disorders [77,78].

Inflammatory markers are consistently reported as elevated in chronic inflammatory skin diseases and mental disorders. The last 5 years’ literature evidenced mainly a relationship between depression and inflammatory processes regarding Ps and AD [25]. Differently, biological mechanisms underlying HS and depression and/or anxiety have not been scrutinized extensively so far, and, in fact, we retrieved no relevant studies on such topic.

Furthermore, based on interventional clinical trials, a few authors suggested that elevated concentrations of proinflammatory cytokines are associated with several chronic skin diseases and concluded that such diseases might be causally linked to the coexistent depressive or anxiety symptoms.

As to the effects of biologics on skin diseases and mental symptoms, available studies do not allow conclusions on whether improvement of mental symptoms are due to the direct anti-inflammatory effect of biologics or to the indirect effect of improved dermatologic disorder. On the other hand, nonsteroidal anti-inflammatory drugs (NSAIDs) have also shown anti-inflammatory and antidepressant effects by inhibiting proinflammatory cytokines [79]. However, other studies did not confirm such results, proving, on the contrary, that NSAIDs administered to patients with comorbid inflammatory diseases and depression may cause severe adverse effects rather than beneficial effects. Moreover, cotreatment of NSAIDs with SSRIs proved to attenuate the effects of the antidepressant drugs [80]. In addition, clinical studies on comorbid AD patients evidenced a reduced 5-HT plasma concentration [57], high 5-HT, 5-HT1A, 5-HT2A receptors, and SERT immunoreactivity in the inflammatory infiltrate of lesional skin [58]. A few immunological mechanisms were also identified, as a reduced NK activity and decreased plasma IL-4 levels, outlined as central mechanisms of the complex immunological framework involved. However, to what extent such mechanisms contribute clarifying the comorbidity presence remains unclear [55]. Vinnik et al. [56], in addition, indicated the increased plasma levels of IgE and cortisol. Interestingly, the authors hypothesized that particularly in AD male patients, the major active neurosteroid DHEA appeared significant as a regulator of IgE synthesis and eosinophil proliferation, besides the increased cortisol levels and the activated HPA axis response caused by the IgE-mediated degranulation of mast cells. In this respect, DHEA may represent a possible link between peripheral organs and the brain, in line with the findings reported by Kaga et al. [53].

Differently, the studies we retrieved on Ps patients suggested a mechanism possibly involved in the comorbidity process, that is, the plasma level decrease of melatonin. However, as no difference arose in the serum melatonin levels when Ps patients were subdivided into two groups, with and without depressive symptoms, melatonin reduction was excluded as a common pathogenetic mechanism involved in Ps and depression [59].

Among immune-mediated mechanisms, the role of IL-17A was highlighted as well in comorbid Ps depressed patients, though indirectly by using a biologic drug (ixekizumab, a high-affinity monoclonal antibody selectively targeting IL-17A). In addition, Griffiths et al. [41] reported the efficacy of such biologic on depression and serum levels of sCRP, suggesting that both systemic inflammatory markers and IL-17A may be involved in Ps comorbid depressive patients. Differently, Marek-Józefowicz et al. [61] reported the increased plasma concentrations of IL-6 as a shared biological mechanism in Ps depressed patients with specific affective temperament dimensions.

On the other hand, Pietrzak et al. [60] reported as shared biological markers both the increased plasma concentrations of IL-18 and low concentrations of 25-hydroxy-vit D_3_ associated with depression severity in men with Ps and concurrent high BMI. Vitamin D is reported to contribute to the pathogenesis of different skin diseases, among which Ps [81]. The epidermis function needs to be considered a natural source of vitamin D synthesis by the sun’s ultraviolet light B (UVB) or other UVB sources. The accumulating evidence shows vitamin D as a key modulator of immune and inflammatory mechanisms [81–83]. However, it remains controversial the effectiveness of supplemented vitamin D as an adjunctive treatment in patients affected by Ps and depression or anxiety [84,85].

Most studies we analyzed showed some limitations in detecting the interrelationships in chronic inflammatory processes.

In particular: (a) mental symptoms comorbid with chronic skin inflammatory disorders are not adequately assessed due to the variability of questionnaires, lack of validated questionnaires specific to dermatologic patients, and limited dermatologists’ familiarity with the psychopathology measurement tools; and (b) studies are generally characterized by small sample sizes, poor psychopathological evaluation (usually self-report inventories), and lack of cognitive functioning and stigma assessment. Furthermore, such studies use heterogeneous indices of inflammation (often one laboratory marker or few cytokines only).

Finally, statistical modeling of the interactions concerning mental and physical symptoms, QoL, and hypothesized underlying mechanisms proved inadequate to determine the complex correlation of the different sets of variables.

## Conclusions

Mental disorders associated with the psychosocial burden of chronic inflammatory skin diseases may impair the response to treatment and negatively affect the physical disease itself which, in turn, may worsen mental symptoms, thus contributing to undermine the patients’ QoL [86–88].

Future research should investigate more deeply the mechanisms that determine the highly frequent comorbidity of mental disorders and chronic skin inflammatory diseases, as well the interrelationships between mental and physical symptoms.

Moreover, dermatologists and psychiatrists should jointly identify the processes based on which proinflammatory cytokines and immune deficiency cause mental symptoms. Proinflammatory cytokines production and cellular immune responses downregulation can thus contribute to prolonged inflammation and delayed healing, as well as to functional decline.

The longitudinal course also needs to be examined to establish the causative interrelationship among all the involved mechanisms. It is still unknown how peripheral mechanisms may involve central brain mechanisms and vice versa, and which biological factors may function as a linking system to trigger macrophages and microglia activation.

Future dermatological studies should be centered on a comprehensive psychopathological evaluation through self- and clinician-rated instruments, including cognitive functioning, stigma, coping strategies, and QoL [81], in addition to plasma levels of inflammatory and tissue markers of subclinical inflammatory damage.

Recognizing more promptly mental disorders in individuals with chronic inflammatory diseases and achieving effective treatments for mental symptoms might significantly improve patients’ health and QoL. Moreover, early detection of mental diseases would enable patients, caregivers, and the health system to reduce costs, particularly the indirect ones [82]. Finally, establishing the reciprocal relationship between mental and physical symptoms would foster specialists’ multidisciplinary activity, which would be beneficial to students, trainees, healthcare professionals, patients, caregivers, relatives, and researchers.

## Data Availability

Data supporting the findings of this study are available in Tables 1–3 and in the Supplementary Material. Furthermore, data are available from the authors on reasonable request.

## Supplementary Materials

To view supplementary material for this article, please visit http://dx.doi.org/10.1192/j.eurpsy.2021.2249. click here to view supplementary material
