# Supplementary material for: A systematic review on shared biological mechanisms of depression and anxiety in comorbidity with psoriasis, atopic dermatitis, and hidradenitis suppurativa
Source: Eur Psychiatry. 2021 Nov 25;64(1):e71. doi: 10.1192/j.eurpsy.2021.2249 (PMC8668448; doi:10.1192/j.eurpsy.2021.2249)
Supplement: Supplementary file 1 [file S0924933821022495sup001.zip › S0924933821022495sup001.doc]

**Supplementary Figure 1.** Flow diagram showing study selection process of included articles searched on PubMed

| **Search terms**  **(MeSH headings)** | (“Biological Factors”[MeSH Terms] OR “Inflammation”[MeSH Terms] OR “Immune System Phenomena”[MeSH Terms] OR “Amino Acids, Peptides, and Proteins”[MeSH Terms]) AND (“Depression”[MeSH Terms] OR “Depressive Disorder”[MeSH Terms] OR “Affective Symptoms”[MeSH Terms] OR “Anxiety Disorders”[MeSH Terms] OR “Anxiety”[MeSH Terms]) AND (“Hidradenitis”[MeSH Terms] OR “Psoriasis”[MeSH Terms] OR “dermatitis, atopic”[MeSH Terms]) AND (“loattrfull text”[Filter] AND “English”[Language]). |
| --- | --- |

Articles identified from electronic searches from inception to May 2021

(n= 111)

Articles excluded after full text review with motivations (n=48)

- Review articles (n=26)
- Case reports/Letters to editor (n=2)
- No reporting on shared mechanisms (n=6)

Duplicates removed

(n=4)

Citations excluded by initial screening of titles and abstracts

(n=59)

Studies included in the present descriptive review

(n= 14)

**1**

[DPI-003]
