# Supplementary material for: A systematic review on shared biological mechanisms of depression and anxiety in comorbidity with psoriasis, atopic dermatitis, and hidradenitis suppurativa
Source: Eur Psychiatry. 2021 Nov 25;64(1):e71. doi: 10.1192/j.eurpsy.2021.2249 (PMC8668448; doi:10.1192/j.eurpsy.2021.2249)
Supplement: Supplementary file 1 [file S0924933821022495sup001.zip › S0924933821022495sup002.doc]

**Supplementary Figure 2.** Flow diagram showing study selection process of included articles searched on Scopus

| **Search terms** | TITLE-ABS-KEY ( ( "Depression" OR "Depressive Disorder" OR "Affective Symptoms" OR "Anxiety Disorders" OR "Anxiety" ) AND ( "Hidradenitis" OR "Psoriasis" OR "dermatitis atopic" ) AND ( "Biological Factors" OR "Inflammation" OR "Immune System Phenomena" OR "Amino Acids" OR peptides OR proteins ) ) AND ( LIMIT-TO ( DOCTYPE , "ar" ) ) AND ( LIMIT-TO ( LANGUAGE , "English" ) ) |
| --- | --- |

Articles identified from electronic searches from inception to May 2021

(n= 323)

Articles excluded after full text review with motivations (n=8)

- No reporting on shared mechanisms (n=7)

Duplicates removed

(already selected on PubMed search) (n=10)

Potentially relevant article ordered for detailed full text screening

(n=18)

Studies included in the present descriptive review

(n= 1)

**1**

[DPI-003]

Citations excluded by initial screening of title and abstracts (n=305)
