# Supplementary material for: A systematic review on shared biological mechanisms of depression and anxiety in comorbidity with psoriasis, atopic dermatitis, and hidradenitis suppurativa
Source: Eur Psychiatry. 2021 Nov 25;64(1):e71. doi: 10.1192/j.eurpsy.2021.2249 (PMC8668448; doi:10.1192/j.eurpsy.2021.2249)
Supplement: Supplementary file 1 [file S0924933821022495sup001.zip › S0924933821022495sup003.doc]

**Supplementary Figure 3.** Flow diagram showing study selection process of included articles searched on APA PsycInfo®

| **Search terms** | (Depression OR Depressive Disorder OR Affective Symptoms OR Anxiety Disorders OR Anxiety) AND (Hidradenitis OR Psoriasis OR dermatitis atopic ) AND (Biological Factors OR Inflammation OR Immune System Phenomena OR Amino Acids OR peptides OR proteins) (Academic journals) NOT (Books AND Dissertations and theses) NOT (Comment/Reply AND Letter) NOT (Systematic revisions AND Clinical cases AND Interviews AND Meta-analyses) English |
| --- | --- |

Articles identified from electronic searches from inception to May 2021

(n= 20)

Articles excluded after full text review with motivations (n=6)

- No reporting on shared mechanisms (n=6)

Duplicates removed

(already selected on PubMed and Scopus search) (n=13)

Potentially relevant article ordered for detailed full text screening

(n=7)

Studies included in the present descriptive review

(n= 1)

**1**

[DPI-003]
