# Supplementary material for: A systematic review on shared biological mechanisms of depression and anxiety in comorbidity with psoriasis, atopic dermatitis, and hidradenitis suppurativa
Source: Eur Psychiatry. 2021 Nov 25;64(1):e71. doi: 10.1192/j.eurpsy.2021.2249 (PMC8668448; doi:10.1192/j.eurpsy.2021.2249)
Supplement: Supplementary file 1 [file S0924933821022495sup001.zip › S0924933821022495sup004.doc]

**Supplementary Table 1.** Risk of bias assessment in non-randomized studies of interventions (NRSI).

| **Authors**  **(year of publication)** | **Type of study** | **Pre-intervention domains** | | **At-intervention domain** | **Post-intervention domains** | | | | **Overall Risk of Bias** |
| --- | --- | --- | --- | --- | --- | --- | --- | --- | --- |
|  |  | ***Confounding bias*** | ***Selection bias*** | ***Information bias*** | ***Confounding bias*** | ***Selection bias*** | ***Information bias*** | ***Reporting bias*** |  |
| Hashiro et al. (1998) | Case-control | No information | No information | High | No information | Low | No information | Low | High |
| Rasul et al. (2016) | Cross-sectional | High | Moderate | Moderate | Moderate | Low | No information | Low | High |
| Vinnik et al. (2020) | Case-control | High | Low | High | Low | Low | No information | Low | High |
| Jaworek et al (2020) | Case-control | Low | Low | High | Low | Low | No information | Low | High |
| Kartha et al. (2013) | Case-control | High | No information | High | No information | Low | No information | Low | High |
| Pietrzak et al. (2018) | Case-control | Low | Low | High | High | Low | No information | Low | High |
| Marek-Józafowicz et al. (2021) | Case-control | Moderate | No information | High | No information | Low | No information | Low | High |

**Supplementary Table 2.** Risk of bias assessment in randomized control studies (RCTs).

| **Authors**  **(year of publication)** | ***Random sequence generation***  ***(selection bias)*** | ***Allocation concealment (selection bias)*** | ***Blinding of participants and personnel (performance bias)*** | ***Blinding of outcome assessment***  ***(detection bias)*** | ***Incomplete outcome data addressed (attrition bias)*** | ***Selective reporting (reporting bias)*** |
| --- | --- | --- | --- | --- | --- | --- |
| Griffiths et al. (2017) | Low | Low | Low | Low | High | Low |
